# Exploring the Use of Indigenous Wild Vegetables by the Basotho People of Southern Africa: A Comprehensive Review of the Literature and Nutritional Analysis of Selected Species

**DOI:** 10.3390/foods12142763

**Published:** 2023-07-20

**Authors:** Rudzani Ralph Tshikororo, Abdulwakeel Ayokun-nun Ajao, Annah Ntsamaeeng Moteetee

**Affiliations:** Department of Botany and Plant Biotechnology, APK Campus, University of Johannesburg, P.O. Box 524, Auckland Park, Johannesburg 2006, South Africa; rudzanitshikororo21@gmail.com (R.R.T.); amoteetee@uj.ac.za (A.N.M.)

**Keywords:** mineral analysis, food security, malnutrition proximate composition, southern Africa, traditional leafy vegetables

## Abstract

Wild indigenous vegetables have recently been receiving attention due to their accessibility and potential to fight malnutrition. The current study investigated the nutritional profile of 10 selected wild indigenous vegetables, namely *Asclepias multicaulis*, *Lepidium africanum*, *Erucastrum austroafricanum*, *Solanum nigrum*, *Sonchus dregeanus*, *Sonchus integrifolius*, *Sonchus nanus*, *Rorippa fluviatilis*, *Tribulus terrestris*, and *Urtica lobulata*, consumed by the Basotho people of southern Africa. This was done by first compiling a comprehensive literature review to identify the knowledge gaps and further analysing the selected vegetables for mineral contents and proximate compositions using standard analytical procedures of AOAC. The literature survey revealed that 90 wild plants are used as vegetables by the Basotho people, and there are knowledge gaps on the nutritional value of many species. Mineral analyses of the wild vegetables showed that *Asclepias multicaulis* and *Sonchus dregeanus* are rich in minerals such as Al, Ca, K, Mg, Na, P, and S and can compete favourably with commercialised vegetables such as lettuce and spinach in terms of mineral components. Also, all the wild vegetables studied have more than 12% recommended caloric protein value except *Tribulus terrestris* (10.07%) and *Lepidium africanum* (11.32%). The crude fat content in *Asclepias multicaulis*, *Lepidium africanum*, *Rorippa fluviatilis*, *Erucastrum austroafricanum*, and *Urtica lobulata* fall within the range required for healthy living. The concentrations of cadmium, copper, and lead in all the vegetables studied are below the detection level, thus making them non-toxic and safe for consumption.

## 1. Introduction

The use of conventional food plants has increased, as they provide nutrients the body needs for energy and the regulation of body processes [1]. Urbanisation, natural disasters, and rising populations negatively impact food security, as most plants cannot meet the demand. This menace has led to food price increases, and, as a result, people in developing countries heavily rely on wild food plants [2,3]. In developing countries such as South Africa, wild plants serve as alternative food, and mostly the leafy parts are used as they contain the bulk of the nutrients [4,5]. Green vegetables were used long before the history of modern man by people such as the Khoisan. For the past 120,000 years, they used wild vegetables and fruits as sources of food and medicine [6]. In South Africa, different tribes use leafy vegetables called *moroho* (Sesotho, Venda) or *imifino* (isiXhosa, isiZulu). The vegetables could be leafy, consisting of young leaves, succulent stems, flowers, and very young fruits [6]. Rural people depend on these vegetables as they are affordable and possess valuable nutrients such as minerals, protein, iron, fibre, and vitamins [7]. In addition, these vegetables contribute to diets and food security in periods of food crisis for humans and livestock [4,6]. Reports have shown that wild vegetables contain high mineral, vitamin, and protein contents, sometimes even higher than cultivated vegetables such as *Brassica juncea* (mustard), *Lactuca sativa* (lettuce), and *Spinacia oleracea* (spinach) [8,9]. Vegetable micronutrients play an important role in body maintenance and compensate for nutrients usually inadequate in regular diets [10]. These nutrients are known for their involvement in body metabolisms, such as bodybuilding and protection against diseases associated with blood circulation and coronary artery failure. Another benefit is their ability to prevent constipation and enhance digestion [11].

Like many cultures in southern Africa and worldwide, the Basotho people in rural areas depend on biological resources for survival, especially fruits and vegetables [12]. The Indigenous use of these vegetables is passed orally from one generation to another, as they are also used for medicinal purposes. For example, *Portulaca oleracea* L. is a wild vegetable traditionally used to prevent skin diseases and premature ageing [13]. The dried leaves of *Taraxacum officinale* F.H. Wigg. are used to produce digestive or diet drinks and herb beers. Additionally, the leaves are used as a diuretic and inflammation modulator [14].

**Figure 1:**
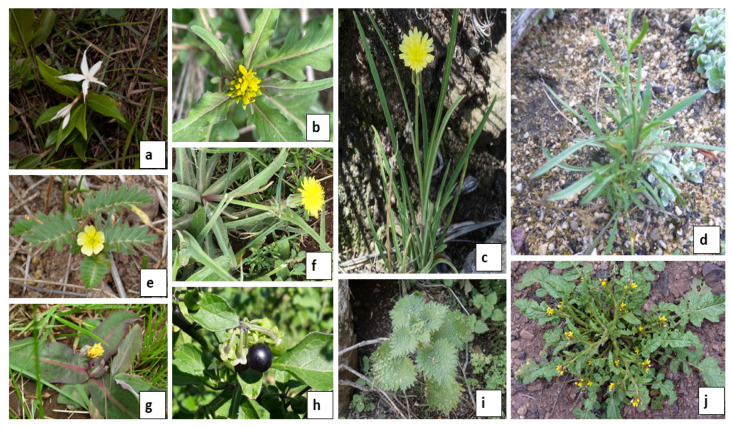
(**a**) *Asclepias multicaulis* (Francoisdu Randt; https://www.ispotnature.org/communities/southern-africa/view/observation/390371/; accessed on 4 July 2023), (**b**) *Rorippa fluviatilis* (Troos van der Merwe; https://www.biodiversity4all.org/observations/101315524; accessed on 4 July 2023), (**c**) *Sonchus integrifolius* (Ansell; https://www.biodiversity4all.org/observations/18301578; accessed on 4 July 2023), (**d**) *Lepidium africanum* subsp. *africanum* (PetraBroddle, https://www.ispotnature.org/view/user/20689; accessed on 4 July 2023), (**e**) *Tribulus terrestris* (Kate Braun; https://ispot.org.za/species_dictionary/Tribulus%20terrestris; accessed on 4 July 2023), (**f**) *Sonchus dregeanus* (David Hoare https://www.inaturalist.org/observations/23041383; accessed on 4 July 2023), (**g**) *Sonchus nanus* (Karen Zunckel; https://www.ispotnature.org/communities/southern-africa/view/observation/333694/sonchus-nanus; accessed on 4 July 2023), (**h**) *Solanum nigrum* (Joanna Lister; https://www.ispotnature.org/communities/southern-africa/view/observation/775755/nastergal-solanum-nigrum; accessed on 4 July 2023), (**i**) *Urtica lobulata* (Mahomed Desai; https://www.inaturalist.org/observations/136404517; accessed on 4 July 2023), (**j**) *Erucastrum austroafricanum* (Nickrent D. L.; http://www.phytoimages.siu.edu/Brassicaceae_Sisymbrium_thellungii_117631.html; accessed on 4 July 2023).

Due to the significance of indigenous vegetables in global food security, the International Conference on Nutrition (ICN) in 1992 and the World Food Summit in 1996 reached a consensus that hunger and malnutrition affecting millions of people can be curbed by cultivating and consuming indigenous vegetables [6]. Therefore, the cultivation of indigenous leafy vegetables is an integral part of farming and food consumption systems in southern Africa, and they are also an essential source of micronutrients [15]. Despite people’s reliance on indigenous vegetables, there is a paucity of information on their value regarding phytochemical properties, minerals, and proximate content. Therefore, this study aims to determine the nutritional properties of selected wild indigenous vegetables used by the Basotho people of the Free State Province of South Africa and Lesotho. This was done by first compiling a comprehensive literature review to identify the knowledge gap and further analysing the vegetables for mineral contents and proximate compositions. This study is imperative to create awareness among the public of the nutritional benefits of consuming these vegetables. Some background information on the selected wild indigenous vegetables studied is summarised as follows (Figure 1).

### 1.1. Asclepias multicaulis (E.Mey.) Schltr. 

*Asclepias multicaulis* is a perennial herb reaching up to 50 m in height. Its leaves are short, triangular, and glabrous at the adaxial surface, and the inflorescences are umbel with six to nine solitary flowers. The species is distributed in Lesotho and South Africa, where it is eaten as a vegetable. In South Africa, it can be found in Eastern Cape, Free State, Gauteng, KwaZulu-Natal, and Mpumalanga Provinces. The conservation status of the species is Least Concern (LC) [16].

### 1.2. Lepidium africanum (Burm.f.) DC. Subsp. africanum

*Lepidium africanum* is a common weed with erect or branched stems up to 100 cm tall. It usually thrives during spring and the beginning of summer. The whole plant is edible, but the leaves are mostly cooked as a vegetable. The leaves are used in folkloric medicine to treat respiratory infections. It is widely distributed in Sub-Saharan Africa and South Africa, occurring in all provinces except the Northern Cape Province. The conservation status of the species is LC [16,17].

### 1.3. Erucastrum austroafricanum Al-Shehbaz & Warwick

*Erucastrum austroafricanum* is an erect herb with robust or branched stems up to 60 cm in height and inflorescent racemes with 5 to 12 flowers. The aqueous extract of the plant has been reported to have high antioxidant activity in an in vitro study [18]. The plant is native to South Africa and Lesotho. In South Africa, it is distributed in Eastern Cape, Free State, Gauteng, KwaZulu-Natal, Limpopo, Mpumalanga, and North West Provinces. The species flowering time is from August to December, and the conservation status is LC [16].

### 1.4. Solanum nigrum *L.*

*Solanum nigrum* is an annual herb with an erect stem up to 1 m in height. The leaf is ovate with a pointed apex, usually up to 10 cm long. The species occurs naturally in North Africa, Europe, and most parts of Asia, and it is introduced in southern Africa. Apart from the plant being used as a vegetable, its extract and isolated compounds have been reported to have ethnomedicinal and pharmacological benefits in treating cancers, dermatological conditions, bacterial infections, etc. [19].

### 1.5. Sonchus dregeanus *DC.*

*Sonchus dregeanus* is a perennial herb with an erect stem up to 90 cm tall. Leaves are usually linear-elliptic or linear-elongate, and can be up to 19 cm long. The Basotho people of southern Africa consume the leaves as vegetables and use them medicinally for skin rashes in children [20]. The species is found in Lesotho, Zimbabwe, and all nine provinces in South Africa. The conservation status is LC [16].

### 1.6. Sonchus integrifolius *Harv.*

*Sonchus integrifolius* is a perennial herb with an erect stem up to 55 cm in height. Basal leaves are usually narrowly oblanceolate, reaching up to 10 cm long; cauline leaves are linear or narrowly elliptic, reaching up to 17 cm long. The Basotho people consume the leaves as a vegetable [20]. The aqueous extract of the plant has been reported to elicit antioxidant activity [21]. The plant is distributed in Eswatini, Lesotho, Mozambique, South Africa (all provinces), and Zimbabwe. The conservation status is LC [16].

### 1.7. Sonchus nanus *Sond.* ex *Harv.*

*Sonchus nanus* is an acaulescent perennial herb with crowded basal rosette leaves up to 8 cm long. The Basotho people usually eat the leaves as a vegetable [20]. It occurs naturally in Eswatini, Lesotho, and South Africa (Free State, Gauteng, KwaZulu-Natal, Limpopo, Mpumalanga, and North West Provinces). The conservation status is LC [16].

### 1.8. Rorippa fluviatilis (E.Mey. ex *Sond.*) *Thell.*

*Rorippa fluviatilis* is an aquatic herb with procumbent stems up to 1 m high. The leaves are usually entire to pinnate, and the inflorescences are racemes. The young plants of *Rorippa fluviatilis* are consumed as a vegetable in South Africa and Lesotho [20]. It is distributed in Botswana, Lesotho, Namibia, South Africa (Eastern Cape, Free State, Gauteng, KwaZulu-Natal, Limpopo, Mpumalanga, Northern Cape, North West Provinces), and Zimbabwe. The conservation is LC [16].

### 1.9. Tribulus terrestris *L.*

*Tribulus terrestris* is an annual plant with silky prostrate stems. Leaves are usually opposite and imparipinnate, with oblong leaflets up to 7 mm long. Traditionally, leaves are used as a vegetable and the whole plant is used as medicine to treat an array of ailments such as dermatological conditions, headache, infertility, erectile dysfunction, rheumatism, and stomatitis [20,22]. It is widely distributed across the African continent, and in South Africa, it occurs in all provinces; its conservation status is LC [16].

### 1.10. Urtica lobulata Blume

*Urtica lobulata* is a perennial herb with dense hairs. The leaves are opposite, broadly ovate, or reniform-ovate, reaching up to 17 cm long. Among Basotho people of southern Africa, especially the Southern Sotho, the plant is used as an ingredient for snake bite remedies and the young leaves are consumed as a vegetable [20,23]. Additionally, the aqueous extract of the plant scavenged free radicals in an in vitro study [18]. It is distributed in South Africa (Free State, KwaZulu-Natal, Cape Provinces) and Lesotho. The conservation status is LC [16].

## 2. Materials and Methods

### 2.1. Literature Survey

Published and unpublished data on indigenous or wild vegetables used by the Basotho people were sourced from online databases such as Google Scholar, Medicine, PubMed, Science Direct, and Scopus using keywords such as Basotho, mineral analysis, proximate composition, wild vegetables, indigenous vegetables, traditional vegetables, etc. Previous reviews by Letšela et al. [12], Moteetee and Van Wyk [24], and Moffett [25] are very useful in documenting the indigenous vegetables used by the Basotho people. 

### 2.2. Selection of Vegetables Used in This Study

Ten wild indigenous vegetables were selected based on availability and the knowledge gap for mineral and proximate composition analyses. These vegetables were collected from various localities in South Africa and identified at the University of Johannesburg Herbarium (JRAU) by the then curator, Professor Annah Moteetee. The species tested are *Asclepias multicaulis* (E.Mey.) Schltr., *Lepidium africanum* (Burm. f.) DC. subsp. *africanum*, *Erucastrum austroafricanum* Al-Shehbaz & Warwick, *Solanum nigrum* L., *Sonchus dregeanus* DC., *Sonchus integrifolius* Harv. var *integrifolius*, *Sonchus nanus* Sond. ex Harv, *Rorippa fluviatilis* (E.Mey. ex Sond.) Thell, *Tribulus terrestris* L., and *Urtica lobulata* Blume.

### 2.3. Processing of the Vegetables Used in this Study

As the Basotho people consume them, the leafy parts of the vegetables were homogenised using a mortar and pestle [26]. The homogenised vegetables were then freeze-dried, except for the samples used to analyse moisture content. 

#### 2.3.1. Microwave Digestion

Microwave digestion (CEM One Touch^TM^ Technology, CEM Technologies, and Charlotte, NC, USA) was used to digest vegetable samples. Briefly, ca. 0.5 g of each vegetable sample was weighed into Teflon tubes (MARSXpress—High Throughput Vessels). They were then mixed with 10 mL of nitric acid (HNO^3^). A blank solution consisting of just the digesting acid (HNO^3^) without a sample was prepared and digested along with the vegetable samples. The temperature conditions of the microwave-digester were as follows; the temperature program was 25–170 °C for the first 10 min and 170–240 °C for another 10 min at 1000 W, followed by immediate ventilation at room temperature for 20 min. The resulting solutions were cooled and made to the mark with Milli-Q water (Millipore, Bedford, MA, USA) in a 50 mL volumetric flask.

#### 2.3.2. Inductively Coupled Plasma Optical Emission Spectrometry (ICPOES) Analysis

The stock and standard working solutions were prepared using ICPOES standard solutions of each metal to be analysed. Mineral contents, namely aluminium (Al), calcium (Ca), iron (Fe), potassium (K), magnesium (Mg), manganese (Mn), phosphorus (P), lead (Pb), selenium (Se), and zinc (Zn) were analysed using concentrations of the samples ranging from 0.1 to 40 mg/mL. The analysis was done in triplicate on ICPOES equipment (Spectro ARCOS, Spectro Analytical Instruments, Kleve, Germany) under the instrumental conditions presented and at standard wavelengths appropriate for foods (Table 1 and Table 2) [27]. Results obtained were expressed as mg/100 g dry weight of the sample.

### 2.4. Proximate Analyses

Proximate analyses were carried out based on dry matter, except for analyses of moisture content.

#### 2.4.1. Moisture Content Determination

Moisture was determined using the procedure described by the American Association of Cereal Chemists (AACC) Method 44—15A (1999). Moisture tins were dried in a forced draught oven at 103 °C for 1 h. The containers were then cooled in a desiccator for about 10 min. Furthermore, the containers were weighed, and 2 g of the sample was poured into the containers and dried in a forced draught oven for 4 h at 103 °C. The samples were cooled for 10 min and weighed. 

The moisture content (%) is calculated as follows:

$$\%\;\mathrm{moisture}=\frac{\left[\left(\mathrm{Mass}\;\mathrm{of}\;\mathrm{food}\;+\mathrm{Mass}\;\mathrm{of}\;\mathrm{tin}\right)-\left(\mathrm{Mass}\;\mathrm{of}\;\mathrm{tin}\right)\right]-\left[\left(\mathrm{Mass}\;\mathrm{of}\;\mathrm{dry}\;\mathrm{food}\;+\mathrm{Mass}\;\mathrm{of}\;\mathrm{tin}\right)-\left(\mathrm{Mass}\;\mathrm{of}\;\mathrm{tin}\right)\right]\;}{\left[\left(\mathrm{Mass}\;\mathrm{of}\;\mathrm{food}+\mathrm{Mass}\;\mathrm{of}\;\mathrm{tin}\right)-\left(\mathrm{Mass}\;\mathrm{of}\;\mathrm{tin}\right)\right]}\times 100$$

#### 2.4.2. Crude Protein Determination

The micro Kjeldahl method described by the Association of Official Analytical Chemists (AOAC 2005) was adopted. First, 2 g of the samples was mixed with 10 mL of concentrated sulphuric acid (H_2_SO_4_) in a heating tube. One tablet of selenium catalyst was added to the tube and then heated inside a fume cupboard. The digested solution was then transferred into a 100 mL volumetric flask and made up with distilled water. The 10 mL portion of the digested solution was then mixed with equal volumes of 45% NaOH solution before pouring into the Kjeldahl distillation apparatus. The mixture was distilled, and the distillate was collected into 4% boric acid solution containing three drops of indicator.

Furthermore, 50 mL of distillates was collected and titrated as well. The experiments were conducted in duplicate to calculate average values. The average nitrogen content was calculated and multiplied by 6.25 to obtain the crude protein content. The nitrogen content was converted to percentage protein by using a protein conversion factor of 6.25.

This was given as:

$$\%\mathrm{Nitrogen}=\frac{\left[\left(100\times \;W\;\times \;N\;\times \;14\times \;\mathrm{Vf}\;\right)T\right]}{100\times Va}$$

where W = weight of the sample, N = normality of the titrate (0.1 N), Vf = total volume of the digest = 100 mL, T = titre value, and *Va* = aliquot volume distilled.

#### 2.4.3. Ash Determination 

Ash is the substance remaining after oxidative combustion of all the organic matter in food. It is, therefore, a measure of the food’s mineral content. Ash content was determined using the AACC method 08—01 (1999). Crucibles were dried in an oven at 100 °C for 5 h and allowed to cool before being weighed; 2 g of samples were then put into the crucibles. The crucibles containing samples were then placed on a tripod and heated until the samples were charred. The charred samples were then placed in a muffle furnace and heated at 550 °C for 5 h. The samples were removed, cooled in a desiccator until reaching room temperature, and weighed. 

The percentage of ash was calculated as follows:

$$\%\;\mathrm{Ash}=\frac{\left(\mathrm{Mass}\;\mathrm{of}\;\mathrm{ash}\;+\;\mathrm{Mass}\;\mathrm{of}\;\mathrm{crucible}\right)-\left(\mathrm{Mass}\;\mathrm{of}\;\mathrm{crucible}\right)\;}{\left(\mathrm{Mass}\;\mathrm{of}\;\mathrm{food}+\mathrm{Mass}\;\mathrm{of}\;\mathrm{cricible}\right)-\left(\mathrm{Mass}\;\mathrm{of}\;\mathrm{crucible}\right)}\times 100$$

#### 2.4.4. Crude Fat Determination

Crude lipid was determined by adopting petroleum ether extract of the sample according to the AACC method 30—25 (1983) using a soxhlet test apparatus. The ether was removed from the collection flask at low-temperature volatilisation before being oven-dried. The residue fat was then dried in the oven at 100 °C for 30 min.

The percentage of fat was calculated using the following formula:

$$\%\;\mathrm{Lipid}=\frac{\left[\left(\mathrm{Mass}\;\mathrm{of}\;\mathrm{beaker}\;+\;\mathrm{Mass}\;\mathrm{of}\;\mathrm{extracted}\;\mathrm{fat}\right)-\left(\mathrm{Mass}\;\mathrm{of}\;\mathrm{beaker}\right)\right]}{\mathrm{Mass}\;\mathrm{of}\;\mathrm{sample}\;}\times 100$$

## 3. Results and Discussion

**Figure 2:**
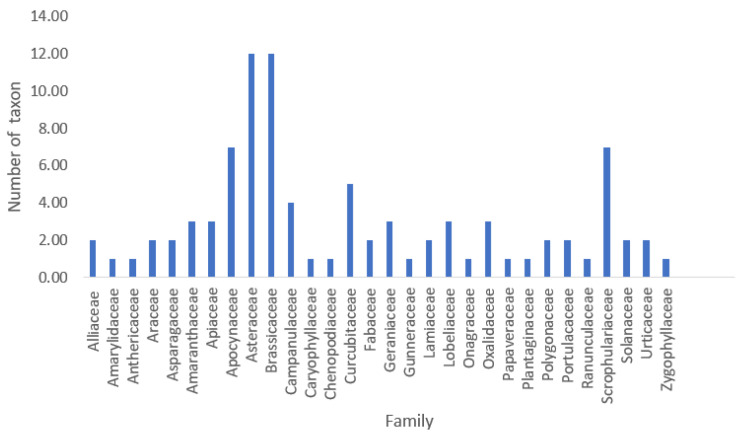
Distribution of wild vegetables used by the Basotho people according to families.

The literature survey revealed that the Basotho people consume 90 species of leafy wild indigenous vegetables belonging to 30 families. The list of species is presented in Table 3. Author citations are included with the name of the vegetables and will not be repeated elsewhere. From the study, the most frequently used families are Brassicaceae and Asteraceae (12 spp each), followed by Apocynaceae and Scrophulariaceae (7 spp each), Cucurbitaceae (5 spp), and Campanulaceae (4 spp). The remaining species are distributed among the remaining 24 families, such as Gunneraceae, Papaveraceae, Ranunculaceae, and Zygophyllaceae (Figure 2). It is important to note that other ethnic groups, such as the Zulu and Xhosa, also consume a vast array of these vegetables in southern Africa. It is unsurprising that the families Asteraceae and Brassicaceae topped the list of the wild indigenous vegetables consumed by the Basotho, as both are known to be food sources. The family Asteraceae is also the largest family of flowering plants, and the most famous vegetables popularly consumed by people around the world belong to this family, such as the common sunflower (*Helianthus annuus* L.), lettuce (*Lactuca sativa* L), and artichokes (*Cynara cardunculus* var. *scolymus* (L.) Benth.). Vegetables belonging to the family Brassicaceae are widely cultivated for domestic consumption and as a source of income by poor citizens in South Africa [28]. The vegetables in this family are also the most studied worldwide, especially *Brassica oleracea* L. The family members contain significant concentrations of dietary nutrients, including glucosinolates, polyphenols, minerals, vitamins, and antioxidant phytonutrients [29].

Apocynaceae, the second most implicated family alongside Scrophulariaceae, with seven species of wild vegetables each, has been reported as the flowering plant family with the highest number of edible plants in southern Africa [30]. For example, species such *Asclepias multicaulis*, *Cynanchum virens*, *Pachycarpus rigidus*, *P. vexillaris*, *Parapodium costatum*, *Riocreuxia torulosa* var. *torulosa*, and *Xysmalobium undulatum* are used as food and medicine by different cultures in southern Africa, including the Basotho [24]. At the generic level, *Nemesia* (5 spp.) is the most speciose genus, followed by *Sonchus* (4 spp.) and *Amaranthus* (3 spp.).

Despite the wide use of plants as vegetables among Basotho, a literature survey revealed that knowledge gaps exist regarding the chemical constituents, mineral composition, and nutritional value of many species used as vegetables by the Basotho people. For instance, 28 species have been evaluated for their chemical constituents, while only 15 of the 90 implicated plants used as vegetables in the region have been assessed for their nutritional value. 

Species such as *Asparagus africanus*, *Centella asiatica*, *Chenopodium album*, *Zantedeschia aethiopicum*, *Tragopogon porrifolius*, *Oxalis corniculata*, and *Portulaca oleracea* are the most studied for phytochemical and nutritional analysis. This is expected as they are consumed by many groups worldwide, not only by Basotho. 

**Table 3:** List of indigenous wild vegetables consumed by Basotho people.

Family	Taxon	Sesotho Name	Parts Used	Phytochemical Data	Nutritional Value
Alliaceae	*Tulbaghia acutiloba* Harv.	Motsuntsunyane	Young plants	No records	No records
	*T. leucantha* Baker	Sefotha-fotha	Young plants	No records	No records
Amarylidaceae	*Cyrtanthus stenanthus* Baker	Lepontoane	Bulb	No records	No records
Anthericaceae	*Chlorophytum fasciculatum* (Baker) Kativu	Lehaohao (ss)	Roots	No records	No records
Araceae	*Zantedeschia aethiopicum* (L.) Spreng	Mothebe	Leaves and stems	Cycloartane, triterpenes and phenylpropanoids [31,32]	No records
	*Z. albomaculata* (Hook.) Baill. subsp. *albomaculata* L.	Mohalalitoe (ss)	Leaves	No records	No records
Asparagaceae	*Asparagus africanus* Lam.	Lelala-tau-le-leholo, or Leunyeli	Young shoots	Steroids and saponins [33,34]	No records
	*Asparagus setaceus* (Kunth) Oberm.	No records	Young shoots	No records	No records
Amaranthaceae	*Amaranthus deflexus* L.	Theepe	Young shoots	No records	No records
	*A. hybridus* L.	Theepe	Young shoots	Alkaloids, flavonoids, saponin, tannins, phenols, hydrocyanic acid and phytic acid [7]	Moisture content, ash content, crude protein, crude lipid, crude fibre, carbohydrate and minerals (Na, Ca, Fe, Mg, P, Z) [7]
	*A. thunbergii* Moq	Theepe-ea-bokone	Young shoots	No records	No records
Apiaceae	*Centella asiatica* (L.) Urb.	Bolila-ba-linku	Leaves	Flavonoids (kaempferol, apigenin, quercetin, catechin, rutin, and naringin), triterpenes and saponins [35]	Vitamin C, vitamin B1, vitamin B2, niacin, Carotene and vitamin A. The total ash, K, Fe, Ca, P, Mg, SO_4_²^−^ and Na [36]
	*Peucedanum magalismontanum* Sond.	Sepaile	Leaves	No records	No records
	*Pimpinella caffra* (Eckl. & Zeyh.) Dietr.	Mohopu	Leaves	No records	No records
Apocynaceae	*Asclepias multicaulis* (E.Mey.) Schltr.	Lenkileng	Leaves	No records	No records
	*Cynanchum virens* (E.Mey.) Dietr.	Morara-oa-moru	Leaves and roots	No records	No records
	*Pachycarpus rigidus* E.Mey.	Phomametsu	Young plants	No records	No records
	*P. vexillaris* E.Mey.	Leshokhoa	Leaves	No records	No records
	*Parapodium costatum* E.Mey.	Sehamela-poli	Leaves	No records	No records
	*Riocreuxia torulosa* Decne. var *torulosa*	Morarana-oa-moru	Leaves	No records	No records
	*Xysmalobium undulatum* (L.) Aiton f.	Leshokhoa	Leaves	No records	No records
Asteraceae	*Athrixia angustissima* DC.	Phefshoane-e-nyenyane	Leaves	No records	No records
	*A. elata* Sond.	Phefshoana-ea-basiea	Leaves	No records	No records
	*A. phylicoides* DC.	Sephomolo	Leaves	Coumarin [37], Flavonoids [38]	No records
	*Dicoma anomala* Sond.	Mohlasetse	Leaves	No record	No records
	*Gazania krebsiana* Less.	Tsikitlana	Leaves	Flavonoids [39]	No records
	*Sonchus dregeanus* DC.	Leharasoana	Leaves	No records	No records
	*S. integrifolius* Harv. var *integrifolius*	Sethokonyane- se- seholo	Leaves	No records	No records
	*S. nanus* Sond. ex Harv.	Sentlokojan	Leaves	No records	No records
	*S. oleraceus* L.	Leshoabe	Leaves	No records	No records
	*Tragopogon porrifolius* L.	Moetse-oa-pere	Young plants	Quercetin, triterpene, saponins, coumarin, and flavonglycoside [40]	Moisture, protein, lipid content, carbohydrate, and fatty acids [41]
	*Taraxacum officinale* F.H.Wigg.	Dandelion (e)	Leaves	Aglycone, coumarins, and flavonoids [42]	Ca, K, Mg, P, Vitamin C and pro-vitamin A [43]
	*Tolpis capensis* (L.) Schultz-Bip.	Fukuthoane	Young plants	No records	No records
Brassicaceae	*Brassica napus* L.	Rapa	Leaves	No records	No records
	*B. rapa* L.	Rapa	Leaves	Flavonoid [44]	No records
	*B. oleracea var oleracea* L.	Kh’abeche	Leaves	No records	No records
	*Erucastrum austroafricanum* Al-Shehbaz & Warwick	Sepatlapatla	Leaves	No records	No records
	*Lepidium africanum* (Burm. f.) DC. subsp. Africanum	Not available	Leaves	No records	No records
	*Nasturtium officinale* W.T. Aiton	Liababa	Leaves	No records	No records
	*Raphanus sativus* L.	Sepaile	Leaves	No records	No records
	*R. nudiuscula* Thell	Papasane	Leaves	No records	No records
	*Rorippa fluviatilis* (E.Mey. ex Sond.) Thell	Liababe	Leaves	No records	No records
	*Sisymbrium capense* Thunb.	Tlhako-ea-khomo	Young plants	No records	Protein, lipid, vitamin C, carotene, and mineral content [45]
	*S. turczaninowii* Sond.	Sentlokojane	Basal leaves	No records	No records
	*Turritis glabra* L.	Lefisoana	Leaves	No records	No records
Campanulaceae	*Wahlenbergia androsacea* A.DC	Tenane	Young plants	No records	No records
	*W. denudata* A.DC	Tename	Young plants	No records	No records
	*W. krebsii* Cham. subsp. *krebsii*	Tenane	Young plants	No records	No records
	*W. undulata* (L.f.) A.DC.	Tenane	Young plants	No records	No records
Caryophyllaceae	*Cerastium capense* Sond.	Mollo-oa-nku	Young plants	No records	No records
Chenopodiaceae	*Chenopodium album* L.	Seruoe	Young plants	β-carotene, lutein. Proanthocyanidins, Kaempferol, flavonoid, and glycosides [46]	Moisture, carbohydrate, crude fibre, protein [45], lipids, vitamin C, beta; carotene, and minerals [47]
Curcubitaceae	*Citrullus lanatus* (Thunb.) Matsum. & Nakai (wild form)	Tjoto	Young leaves	No records	No records
	*Coccina sessilifilia* (Sond.) Cogn.	Borobahlolo	Leaves	No records	No records
	*Cucumis myriocarpus* Naud.	Monyaku	Leaves	Flavone C-glycosides [48]	Calcium, iron, potassium, and phosphorus [49]
	*Langenaria siceraria* (Mol.) Standl.	Sehoana	Leaves	Flavonoids, saponins, and tannins [50]	Protein and carbohydrates [50]
	*Momordica balsamina* L.	Moholu	Leaves	Alkaloids, flavonoids, glycosides, steroids, terpenes, cardiac glycoside, and saponins [51]	Potassium, magnesium, phosphorus, calcium, sodium, zinc, manganese, and iron [51]
Fabaceae	*Trifolium africanum* Ser. var. *africanum*	Moqopolla-thupa	Inflorescences	No records	No records
	*T. burchellianum* Ser.	Moroko	Inflorescences	No records	No records
Geraniaceae	*Geranium incanum* Burm.f.	Ngope setsoha	Leaves	Tannins, steroidal saponin and flavonoids [52]	No records
	*G. multisectum* N.E.Br.	Bolila-ba-likhomo	Leaves	No records	No records
	*Pelargonium bowkeri* Harv.	Khoara	Leaves	No records	No records
Gunneraceae	*Gunnera perpensa* L.	Qobo	Stems, flower stalks, and leaves	Alkaloids, flavonoids, flavonols, phenols, proanthocyanidins, and tannins [53]	No records
Lamiaceae	*Mentha aquatica* L.	Koena-e-nvenyane	Leaves	No records	No records
	*M longifolia* L.	Koena	Leaves	Flavonoids and monoterpenes [54]	No records
Lobeliaceae	*Lobelia erinus* L.	Lenkoto	Young plants	No records	No records
	*L. preslii* A.DC.	Mahlo-a-konyana	Young plants	No records	No records
	*Monopsis decipiens* (Sond.) Thulin	Malana-a-konyana	Young plants	No records	No records
Onagraceae	*Epilobium hirsutum* L.	Letsoai-la-balisana	Leaves	Phenolic acids, flavonoids, and tannins [55]	No records
Oxalidaceae	*Oxalis corniculata* L.	Bolila-ba-thaba	Whole plant	Phytosterols, phenolic compounds/tannins, and flavonoids [56]	Moisture, carbohydrate, crude protein, crude lipid, Na, Ca, N and Mg [57]
	*O. semiloba* Sond. subps. *semiloba*	Bolila	Leaves and roots	No records	No records
	*O. setosa* E.Mey. ex Sond.	Bolila	Leaves	No records	No records
Papaveraceae	*Papaver aculeatum* Thunb.	Sehlohlo	Young plants	Aporphine alkaloid [58]	No records
Plantaginaceae	*Plantago major* L.	Bolila-ba-Iipoli	Leaves and roots	Flavonoids, alkaloids, iridoid glycosides, and terpenoids [59]	Polysaccharides mucilage, silicic acid, zinc, silica, and potassium [59]
Polygonaceae	*Rumex sagittatus* Thunb.	Bolila-bo-boholo	Leaves	Alkaloids, saponins, and phytate [60]	Crude protein, lipids, fibre, carbohydrates, minerals (Ca, Cu, Fe, K, Mg, P, Mn, N, Na and Zn) [60]
	*R. woodii* N.E.Br.	Bolila-ba-likhomo	Leaves	No records	No records
Portulacaceae	*Portulaca oleracea* L.	Selêlê	Leaves and stems	Alkaloids (oleraceins A, B, C, D and E) [61]. Flavonoids (kaempferol apigenin, myricetin, quercetin and luteolin) [62]	Ca, K, Mg, carbohydrates, and omega-3 fatty acids [63,64]
	*Talinum caffrum* (Thunb.) Eckl. & Zeyh.	Khutsana	Leaves	Alkaloids, polyphenols, saponins, and tannins [65]	No records
Ranunculaceae	*Thalictrum minus* L.	Lefokotsane	Young plants	Alkaloid, triterpenoid and saponin [66]	No records
Scrophulariaceae	*Nemesia albiflora* N.E.Br.	Malana-a-konyana	Young plants	No records	No records
	*N*. *caerulea* Hiern	Malana-a-konyana	Young plants	No records	No records
	*N*. *fruticans* (Thunb.) Benth.	Malana-a-konyana	Young plants	No records	No records
	*N*. *floribunda* Lehm.	Malana-a-konyana	Young plants	No records	No records
	*N*. *pubescens* Benth	Malana-a-konyana	Young plants	No records	No records
	*N*. *rupicola* Hilliard	Malana-a-konyana	Young plants	No records	No records
	*Zaluzianskya peduncularis* Walp.	Emèmèè	Young plants	No records	No records
Solanaceae	*Solanum nigrum* L.	Seshoa-bohloko	Leaves	Alkaloids, terpenoids, flavonoids, saponins, steroids and phenol [67]	Na, K, Ca, Mg, Fe, P, and Zn [67]
	*S*. *retroflexum* Dun.	Seshoa-bohloko	Leaves	No records	No records
Urticaceae	*Urtica dioica* L.	Bobatsi	Young leaves	Alkaloids and saponin [68]	Minerals (especially iron), vitamin C and pro-vitamin A, carotenoids, and fatty acids [69]
	*U. lobulata* Blume.	Bobatsi	Young leaves	Flavonol, proanthocyanidin, and phenolics [70]	No records
Zygophyllaceae	*Tribulus terrestris* L.	Tsehlo	Leaves	Alkaloids, tannins, saponins and cardiac glycosides [71]	No records

### 3.1. Mineral Analyses

In general, it was observed that the mineral contents of the wild vegetables varied considerably. The mineral contents of selected wild vegetables used by the Basotho people are presented in Table 4. Based on aluminium content, the highest concentration was observed in *Sonchus dregeanus* (110.04 mg/100 g), followed by *Asclepias multicaulis* (105.09 mg/100 g), while *Erucastrum austroafricanum* had the lowest concentration (70.96 mg/100 g). The aluminium content observed in all the wild vegetables in this study is higher than what was observed in *Gonostegia hirta* (10.37 mg/100 g), a wild vegetable from China [72]. The result showed that these Basotho wild vegetables are a good source of aluminium and can also supply daily intake of Al in humans [73]. It is important to note that aluminium is required in small quantities in the body, as it is toxic when consumed in high concentrations. According to FAO and WHO [74], the tolerable weekly intake of aluminium is 2 mg/kg bw/wk. The element is crucial for endocrine system metabolism and regulation of digestive enzyme activity.

Asclepias *multicaulis* (641.48 mg/100 g) had the highest concentration of Ca, followed by *Sonchus dregeanus* (574.86 mg/100 g); *Sonchus nanus* had the lowest concentration, at 23.29 mg/100 g. Previous reports revealed that the calcium content range of wild vegetables and wild food plants ranges from 98.32 mg to 194.98 mg/100 g and 27.0 mg to 75 mg/100 g, respectively, which means some of the wild vegetables in the study can compete favourably with wild vegetables and food in terms of calcium [75,76,77,78]. The calcium contents in the wild vegetables consumed by the Basotho, especially *Asclepias multicaulis*, *Sonchus dregeanus*, *Sonchus integrifolius*, *Solanum nigrum*, *Rorippa fluviatilis*, are higher than those of popular commercial vegetables, namely lettuce (36 mg/100 g), mustard (63 mg/100 g), and spinach (99 mg/100 g) [79]. In adults, the daily recommended intake for Ca is 1000 mg [80]. Calcium is essential for muscular movement, the transmission of neuron messages from the brain to the body, and blood coagulation [3].

The concentrations of iron in the wild vegetables studied are very low. The Fe levels in all the vegetables are less than 1 mg/100 g except for *Asclepias multicaulis*, with 1.01 mg/100 g. Previous studies on different traditional vegetables from southern Angola and China have reported much higher Fe concentrations [64,70]. The iron concentration in all the vegetables studied is also less than that of spinach (2.71 mg) [79]. The recommended intake for Fe in adults is between 8 and 18 mg per day [81]. Iron is required for body growth and development. It is also involved in the formation of haemoglobin, myoglobin, and hormones.

The highest content of K was observed in *Sonchus dregeanus* (2945.86 mg/100 g), followed by *Asclepias multicaulis* (1157.58 mg/100 g), whereas the lowest was detected in *Tribulus terrestri**s*** (97.22/100 g). The K content in the studied wild leafy vegetables is relatively high compared to what was reported from other wild vegetables, namely *Amaranthus hybridus*, *Bidens pilosa*, and *Galinsoga parviflora* from southern Angola (287 to 445.51 mg/100 g) [78]. According to the National Institutes of Health (NIH) Office of Dietary Supplements (ODS) [82], the daily recommended intake for K is 2600 mg and 3400 mg for female and male adults, respectively, which means 100 g of *Sonchus dregeanus* can adequately supply the daily recommended intake for potassium, especially in female adults. Potassium is essential for muscle contraction and maintaining blood pressure and cellular fluid.

**Table 4:** Results of the mineral analyses of selected wild vegetables used by the Basotho people.

Mineral Elements	*Asclepias multicaulis*	*Lepidium* *africanum*	*Erucastrum austroafricanum*	*Solanum nigrum*	*Sonchus dregeanus*	*Sonchus* *integrifolius*	*Sonchus nanus*	*Rorippa fluviatilis*	*Tribulus terrestris*	*Urtica lobulata*	*Lactuca sativa*	*Brassica juncea*	*Spinacia oleracea*
Al	105.09± 3.34	71.23 ± 0.93	70.96 ± 1.15	80.85 ± 1.73	110. 04 ± 2.6	85.14 ± 2.32	71.74 ± 1.17	82.24 ± 2.02	80.22 ± 1.61	94.61± 2.90	NA	NA	NA
Ca	641.48 ± 11.80	48.67 ± 2.20	32.04 ± 0.77	209.44 ± 5.30	574. ± 7.10	142.22 ± 3.91	23.29 ± 0.50	121.18 ± 2.70	35.38 ± 2.40	309.14 ± 2.15	36.00	63.00	99.00
Cd	BDU	BDU	BDU	BDU	BDU	BDU	BDU	BDU	BDU	BDU			
Cu	BDU	BDU	BDU	BDU	BDU	BDU	BDU	BDU	BDU	BDU	0.029	0.074	0.13
Fe	1.01 ± 0.00	BDU	0.74 ± 0.01	0.39 ± 0.01	1.59 ± 0.02	0.75 ± 0.01	0.071 ± 0.00	0.39 ± 0.01	BDU	0.098 ± 0.00	0.86	1.61	2.71
K	1157.58 ± 17.70	301.64 ± 4.80	725 ± 11.90	498.52 ± 4.0	2945.86 ± 30.2	727.47 ± 10.40	570.6 ± 5.3	354.5 ± 6.60	97.22 ± 2.90	1081.31 ± 18.23	194.00	152.00	558.00
Mg	216.7 ± 8.4	33.22 ± 1.05	80.35 ± 1.1	90.67 ± 2.5	313.18 ± 8.1	70.48 ± 3.7	13.78 ± 1.1	55.96 ± 3.47	15.86 ± 0.18	149.96 ± 2.5	13.00	48.00	79.00
Mn	0.75 ± 001	BDU	0.38 ± 0.01	0.55 ± 0.06	1.74 ± 0.04	0.36 ± 0.01	0.038 ± 0.00	0.1 + 0. 00	BDU	0.31 ± 0.01	0.25	0.422	0.897
Na	59.57 ± 2.20	4.89 ± 0.02	9.76 ± 0.1	85. 75 ± 3.	92.12 ± 3.62	39.61 ± 2.7	BDU	16.49 ± 1.45	BDU	61.7 ± 2.8	28.00	1100.00	79.00
P	159.68 ± 1.30	56.21± 1.10	103.38 ± 4.10	44.68 ± 3.70	150.08 ± 4.30	90.7 ± 2.10	54.05 ± 2.31	23.8 ± 0.73	6.06 ± 0.10	68. 96 ± 1.90	29.00	108.00	49.00
Pb	BDU	BDU	BDU	BDU	BDU	BDU	BDU	BDU	BDU	BDU	NA	NA	NA
S	623.7 ± 9.40	87.31 ± 2.0	69.07 ± 2.50	161.9 ± 4. 10	288.43 ± 3.60	183.36 ± 2.70	44.28 ± 3.60	29.65 ± 0.90	BDU	BDU	NA	NA	NA
Se	BDU	BDU	BDU	BDU	BDU	BDU	BDU	BDU	BDU	BDU	0.0006	0.0335	0.001
Zn	2.93 + 005	0.75 ± 0.01	1.27 ± 0.05	1.6 ± 0. 0.05	46.7 ± 2.37	1.77 ± 0.03	0.52 ± 0.01	1.23 ± 0.01	0.62 ± 0.01	2.81 ± 0.05	0.18	0.64	0.53

Values are expressed as means in mg/100 g ± standard deviations (*n* = 3). BDU: below detected unit; NA: information not available. The mineral composition of *Brassica juncea*, *Lactuca sativa*, *and Spinacia oleracea* is according to the USDA [79].

Regarding magnesium composition, *Sonchus dregeanus* contains the highest concentration (313.18 mg/100 g), followed by *Asclepias multicaulis* (216.7 mg/100 g), while the lowest was observed in *Sonchus nanus* (13.78 mg/100 g). Apart from the fact that most of the wild vegetables in the study, except for *Sonchus nanus* (13.78 mg/100 g), had higher magnesium contents than wild vegetables from southern Angola [78], *Sonchus dregeanus* and *Asclepias multicaulis* also have higher Mg content than the common commercial leafy vegetables, namely *Lactuca sativa* (lettuce), *Brassica juncea* (mustard), and *Spinacia oleracea* (spinach) with 13 mg/100 g, 48 mg/100 g, and 79 mg/100 g, respectively [79]. The daily recommended intake for Mg in adults is 320 to 420 mg [83]. Magnesium is essential for metabolic reactions in the body and vital for cardiovascular and nervous activities [3].

Similar to Fe, the concentrations of Mn are very low. The Mn levels in all the vegetables are less than 1 mg/100 g, with the exception of *Sonchus integrifolius*, with 1.74 mg/100. However, Mn was not detected in *Lepidium africanum* and *Tribulus terrestris*. The concentrations of Mn reported from the wild vegetables from southern Angola are also less than 1 mg/100 g, while those from KwaZulu-Natal Province in South Africa are greater than 1 mg/100 g [63,78]. When comparing all the wild vegetables in the study with spinach (0.897 mg/100 g), only *Sonchus integrifolius* had a higher concentration of Mn. The daily recommended intake of Mn is between 1.8 mg and 2.3 mg, which means *Sonchus integrifolius* is a good source of Mn for humans [82]. Manganese is essential in the formation of bones, connective tissues, and sex hormones. It also plays a critical role in metabolism, regulation of the body and blood sugar, and calcium absorption.

The results showed that *Sonchus dregeanus*, *Solanum nigrum*, and *Asclepias multicaulis* had higher levels of Na (92.12 mg/100 g, 85. 75 mg/100 g, and 59.57 mg/100 g, respectively). However, Na was not detected in *Sonchus nanus* and *Tribulus terrestris*. The sodium contents of wild vegetables in the study are higher than those of the wild vegetables from Angola (2.7 to 4.9 mg/100 g). The Na contents in *Sonchus dregeanus* and *Solanum nigrum* are also relatively higher than that of spinach (79 mg/100 g) [79]. However, the traditional vegetables from KwaZulu-Natal Province showed a much higher concentration of Na compared to wild vegetables in this study, especially in *Asystasia gangetica* and *Oxygonnum sinuatum*, with 933 mg/100 g and 1460 mg/100 g concentrations, respectively [63]. The recommended daily intake for Na is less than 2300 mg. Sodium is essential for the transmission of nerve impulses, as well as the contraction and relaxation of the muscular system. It also helps in bodily maintenance and water and mineral balancing in the body.

Phosphorus content in the selected wild vegetables consumed by the Basotho people also varied. *Asclepias multicaulis* had the highest concentration (159.68 mg/100 g), closely followed by *Sonchus dregeanus* (150.08 mg/100 g), while *Tribulus terrestris* had the lowest concentration (6.06 mg/100 g). Most wild indigenous vegetables studied, except for *Solanum nigrum* and *Rorippa fluviatilis*, have relatively higher phosphorus contents than the wild leafy vegetables from southern Angola (phosphorus contents range from 35.53 to 52.62 mg/100 g), lettuce (29 mg/100 g), and spinach (49 mg/100 g) [79]. However, the traditional vegetables from KwaZulu-Natal Province, especially *Amaranthus dubius*, *Amaranthus hybridus*, and *Asystasia gangetica*, had higher concentrations (604, 629, 814 mg/100 g) [63]. *Asclepias multicaulis* and *Sonchus dregeanus* can easily meet the daily recommended intake (700 mg) of P in adults if consumed in large quantities. Phosphorus is involved in teeth and bone formation and plays a role in carbohydrate and fat metabolism and the repair and maintenance of cells and tissues.

*Asclepias multicaulis* had the highest concentration of S (623.7 mg/100 g), followed by *Sonchus dregeanus* (288.43 mg/100 g), while *Rorippa fluviatilis* had the lowest concentration (29.5 mg/100 g). However, S was not detected in *Tribulus terrestris* and *Urtica lobulata*. All the wild vegetables in the study have a relatively higher concentration of sulphur when compared to wild indigenous vegetables from southern Angola, with S concentrations ranging from 24.38 mg/100 g to 28.88 mg/100 g [78]. Additionally, the S contents in *Asclepias multicaulis*, *Sonchus dregeanus*, and *Sonchus integrifolius* are higher than broccoli (140 mg/100 g). Currently, there is no recommended dietary intake for S in humans [82]. Sulphur is essential for the building and repair of DNA cells, is involved in the metabolism of food in the body, and contributes to skin and muscle health.

Regarding the Zn concentrations of the wild vegetables studied, the highest concentration was observed in *Sonchus dregeanus* (46.7 mg/100 g), while other vegetables have less than 3 mg/100 g. A previous study on the traditional vegetables consumed in KwaZulu-Natal Province showed higher concentrations of Zn in wild vegetables, especially in *Chenopodium album*, *Amaranthus dubius*, and *Emex australis* (109 mg/100 g, 56 mg/100 g, 20 mg/100 g, respectively) [63]. However, Zn concentrations in this study are slightly higher than the wild indigenous vegetables from southern Angola (0.33 to 0.44 mg/100 g) [78]. Except for *Sonchus nanus and Tribulus terrestris*, all the wild indigenous vegetables in the study have higher Zn concentrations than lettuce (0.18 mg/100 g), mustard (0.64 mg/100 g), and spinach (0.53 mg/100 g). The daily recommended intake for Zn in adults is 40 mg [82]. Zinc is essential for the normal functioning of body systems and promotes immune system functioning, wound healing, and thyroid function.

Copper (Cu) was not detected in any of the vegetables studied. Copper (Cu) is vital for body metabolism, as it serves as a cofactor for enzymes that drive oxidation-reduction reactions and haematopoiesis in the human system [84]. According to European Food Safety Authority [85], the recommended intake for copper is 5 mg per day for adults.

Selenium was not detected in any of the vegetables studied. Se is required to maintain the metabolism of the human body. However, consumption of selenium greater than 400 μg/day may be toxic to the human body [86,87].

The concentrations of cadmium (Cd) and lead (Pb) were also measured in all the wild vegetables to detect the safety of the vegetables when consumed. Both Cd and Pb were below detection units for all vegetables tested in the study. High concentrations of these elements can be toxic as they are heavy metals. Intake of Cd can cause hepatic and renal dysfunction, joint and bone degeneration, and blood damage [88]. Ingestion of lead can cause high blood pressure and renal dysfunction [89].

### 3.2. Proximate Composition

In general, it was observed that the proximate composition of the wild vegetables varied considerably. The results of proximate analyses of the wild vegetable are presented in Table 5. The protein content of the wild vegetables showed that *Solanum nigrum* had the highest concentration (25.34%), followed by *Erucastrum austroafricanum* (24.04%), while *Asclepias multicaulis* had the lowest protein content (12.99%). All the wild vegetables studied are good sources of protein except for *Tribulus terrestris* (10.07%) and *Lepidium africanum* (11.32%), with less than 12% of the recommended concentration [90]. Protein is essential for the formation and maintenance of body tissues, enzymes, and hormones. It is also involved in developing antibodies, energy metabolism, and growth.

The crude fat content of the wild vegetables ranged from 0.85% to 4.29%, and the highest concentration was found in *Solanum nigrum* (4.92%), followed by *Tribulus terrestris* (4.27%). *Asclepias multicaulis*, *Lepidium africanum*, *Rorippa fluviatilis*, *Erucastrum austroafricanum*, and *Urtica lobulata*, had concentrations of crude fat ranging from 0.85% to 1.57%. Studies have suggested that 1–2% of caloric fat is desirable for healthy living, as excess fat intake can lead to obesity, which could cause cardiovascular ailments and cancers [91,92]. Crude fat plays an important role in the production of energy that drives body metabolism and protects the integrity of the cell membrane and adipose tissues [93].

Regarding the ash content, the wild vegetables contain appreciable quantities of ash, with the highest concentration found *Urtica lobulata* (21.53%), followed by *Rorippa fluviatilis* (20.7%), and the lowest concentration found in *Sonchus integrifolius* (4.63%). The ash contents of most vegetables studied are relatively higher than those reported from Bangladesh [90]. The highest quantity of ash was found to be 13.26% in *Dryopteris filix-mas.* Additionally, all the wild vegetables studied contain more ash than those reported for *Heinsia crinita* (3.71%) and *Lasianthera africana* (2.71%), which are two indigenous vegetables consumed in Akwa Ibom State, Nigeria [94]. Ash is essential for promoting antioxidative, anti-inflammatory, diuretic, and depurative activities of the body system.

The moisture content of food has been described as a good source of water that hydrolyses the body tissue during metabolism [90]. In general, the wild vegetables contain appreciable moisture content in that the highest concentration was found in *Sonchus dregeanus* (96.03%), followed by *Solanum nigrum* (93.04%), and the lowest in *Tribulus terrestris* (52.71%). These results showed that *Sonchus dregeanus* is a good source of moisture in that the moisture content found in the vegetable is considerably higher than those previously reported in the vegetables from Pakistan, Nigeria, Bangladesh, and China [72,90,95,96]. The relatively high moisture content in all the vegetables studied implies that they may have a short shelf life and high microbial contamination if not well preserved.

Despite the global progress in food security, the problem of hunger and essential micronutrient deficiency among people in African countries, including southern Africa, is a major concern [97]. This problem might be due to inadequate consumption of fruit and vegetables [98]. Wild indigenous vegetables listed and analysed in this study could find relevance in solving the problem of malnutrition and hunger if adequately harnessed. Apart from the fact that wild indigenous vegetables can provide a cheaper source of nutrients to combat malnutrition among locals when consumed alone or as a salad or relish with starch staple dishes [99], local production and promotion of these wild vegetables at small scale can also provide a source of income for poor people in the community, as well as direct access for harvesting.

## 4. Conclusions

The present article attempts to identify the knowledge gap by conducting a literature survey regarding the nutritional composition of wild indigenous vegetables consumed by Basotho people and further analysing some selected vegetables for mineral contents and proximate compositions. Results obtained from the literature survey revealed that 90 plants are used as wild vegetables by Basotho people, and there are knowledge gaps on the chemical constituents, mineral composition, and nutritional value of many species implicated in this study. Only 28 species have been evaluated for their chemical constituents, while 15 have been assessed for their nutritional compositions. Mineral analyses of the wild vegetables showed that *Asclepias multicaulis* and *Sonchus dregeanus* are rich in minerals such as Al, Ca, K, Mg, Na, P, and S and can compete favourably with commercialised vegetables such as lettuce, mustard, and spinach in terms of mineral components. Regarding proximate compositions, all the wild vegetables studied have more than 12% of the recommended caloric protein value except *Tribulus terrestris* (10.07%) and *Lepidium africanum.* The crude fat contents in *Asclepias multicaulis*, *Lepidium africanum*, *Rorippa fluviatilis*, *Erucastrum austroafricanum*, and *Urtica lobulata* fall within the range required for healthy living. *Urtica lobulata* and *Rorippa fluviatilis* have high ash contents, suggesting that the two vegetables are nutrient-rich. *Tribulus terrestris* may have a long shelf life and low microbial infestation due to considerably lower moisture content compared to other vegetables. The concentrations of cadmium, copper, and lead in all the vegetables studied are below the detection level, thus making the vegetables non-toxic and safe for consumption.

Overall, this study has once again confirmed that indigenous wild vegetables are rich in nutrients and minerals and can provide an alternative nutrient source for the locals and poor people to promote food security and alleviate poverty. Future studies should focus on analysing the vegetables for other nutritional contents, such as carbohydrates, crude fibre, and amino acids, and assess the effect of cooking on the concentration of nutrients in the wild vegetables, so they can be harnessed to fight malnutrition. Having ascertained the nutrition prowess of these indigenous vegetables, efforts should also be geared towards their domestication, promotion, and mass propagation so they can be readily available for commercialisation.

## Figures and Tables

**Table 1:** ICPOES working conditions.

Parameters	Conditions
RF power (emission intensity)	1200 W
Nebuliser type	Concentric
Nebulizer flow	0.5 L/min
Gas (as 600 kpa)	Argon
Plasma gas flow	10 L/min
Auxiliary gas flow	0.5 L/min
PMT volts	600 V
Sample flow	0.9 mL/min
Rinse time	5 min

**Table 2:** Element Detection Wavelength (nm) According to Nolte [27].

Element	Wavelength (nm)
Al	396.152
Ca	317.933
Cr	267.716
Cd	228.802
Cu	327.396
Fe	259.940
K	766.490
Mg	285.213
Mn	257.610
Na	589.592
P	178.287
Pb	220.353
S	182.037
Se	196.026
Zn	206.200

**Table 5:** Proximate analysis of wild vegetables.

Wild Vegetables	Protein Content	Crude Fat Content	Ash Content	%Moisture Content (FW)
*Asclepias multicaulis*	12.99 ± 0.05	1.57 ± 0.01	16.38 ± 0.02	92.69 ± 0.23
*Lepidium africanum*	11.32 ± 012	0.85± 0. 03	11.43 ± 0.07	85.25 ± 0.01
*Erucastrum austroafricanum*	24.04 ± 0.01	0.97 ± 0.01	13.89 ± 0.6	73.17 ± 0.20
*Solanum nigrum*	25.34 ± 0.02	4.29 ± 0.10	6.56 ± 0.60	93.04 ± 0.11
*Sonchus dregeanus*	21.49 ± 0.85	2.18 ± 0.06	4.93 ± 0.11	96.03 ± 0.22
*Sonchus integrifolius*	15.07 ± 0.20	2.36 ± 0.01	4.63 ± 0.21	90.85 ± 0.06
*Sonchus nanus*	18.94 ± 0.05	4.26 ± 0.09	7.3 ± 0.05	82.04 ± 0.90
*Rorippa fluviatilis*	21.35 ± 0.11	1.89 ± 0.21	20.7 ± 0.9	86.4 ± 0.50
*Tribulus terrestris*	10.07 ± 0.02	4.27± 0.55	5.94 ± 0.39	52.71 ± 0.01
*Urtica lobulata*	17.29 ± 0.01	1.62 ± 0.13	21.53 ± 0.5	76.85 ± 0.01

Values are expressed as means in percentage (%) ± standard deviations (*n* = 3).

## Data Availability

Data is contained within the article.
